# Digoxin protects against intervertebral disc degeneration via TNF/NF-κB and LRP4 signaling

**DOI:** 10.3389/fimmu.2023.1251517

**Published:** 2023-09-18

**Authors:** Qunbo Meng, Kaiwen Liu, Zhenchuan Liu, Jinbo Liu, Ziyu Tian, Shanshan Qin, Jianlu Wei, Lei Cheng

**Affiliations:** ^1^ Department of Orthopaedic Surgery, Qilu Hospital, Cheeloo College of Medicine, Shandong University, Jinan, Shandong, China; ^2^ Qilu Hospital of Shandong University, Shandong University, Jinan, Shandong, China; ^3^ Department of Radiology, Qilu Hospital of Shandong University, Jinan, Shandong, China; ^4^ Qilu Hospital of Shandong University Spine and Spinal Cord Disease Research Center-International Chinese Musculoskeletal Research Society (ICMRS) Collaborating Center for Orthopaedic Translational Research, Shandong University, Jinan, Shandong, China

**Keywords:** digoxin, intervertebral disc degeneration, TNF, LRP4, inflammation

## Abstract

**Background:**

Intervertebral disc degeneration (IVDD) is a leading cause of low back pain (LBP). The pathological process of IVDD is associated with inflammatory reactions and extracellular matrix (ECM) disorders. Digoxin is widely used for treating heart failure, and it has been reported to have anti-inflammatory effects.

**Objective:**

This study is to investigate the role of digoxin in the pathogenesis of intervertebral disc degeneration as well as the involved molecular mechanism, particularly the potential target protein.

**Methods:**

We exploited a rat needle model to investigate digoxin’s role in intervertebral disc degeneration *in vivo*. Safranin O staining was used to measure cartilaginous tissue in the intervertebral disc. The morphological changes of intervertebral discs in animal models were determined by Hematoxylin-Eosin (H&E) staining and the pathological score. Primary nucleus pulposus cells (NP cells) from intervertebral discs of patients and murine were used in the present study. Western-Blotting assay, Real-time PCR assay, immunofluorescence staining, and immunochemistry were used to detect the role of digoxin in anti-TNF-α-induced inflammatory effects *in vitro*. Transfection of siRNA was used to regulate low-density lipoprotein receptor-related protein 4 (LRP4) expression in NP cells to investigate the potential protein target of digoxin.

**Results:**

Digoxin protected against intervertebral disc degeneration in rat needle models. Digoxin was found to exert its disc-protective effects through at least three different pathways by a) suppressing TNF-α-induced inflammation, b) attenuating ECM destruction, c) significantly promoting ECM anabolism. Additionally, LRP4 was found to be the downstream molecule of digoxin in NP cells for anti-inflammation and regulation of ECM metabolism. The knockdown of LRP4 downregulated the protective effect of digoxin in NP cells.

**Conclusion:**

These findings suggest that digoxin may be a potential therapeutic agent for intervertebral disc degeneration through anti-catabolism and pro-anabolism. Digoxin might also work as an alternative for other inflammation-related diseases.

## 1 Introduction

Low back pain (LBP) is a widespread global health problem, a cause of long-term health problems for people across different incomes levels and countries (1, 2). According to the Global Burden of Disease Study of 2019 (GBD 2019), LBP was the leading cause of years lived with disability (YLDs) worldwide, accounting for about 7.4% (range: 6.2%–8.7%) of the total global YLDs. This translates to a staggering figure of approximately 63.7 million YLDs attributable to LBP. In Europe, the aggregate costs associated with LBP equate to about 0.1%–2% of the gross domestic product. Intriguingly, more than 80% of the total expenditure related to LBP is constituted by indirect costs, including productivity losses and disability-related disbursements (3, 4).

Intervertebral disc degeneration (IVDD) is a complication of LBP (2, 5). A healthy intervertebral disc is composed of a centrally located nucleus pulposus (NP) surrounded by an annulus fibrosus (AF) (6). As the degenerative process advances, particularly with the degeneration of the NP, the intervertebral disc may exhibit a marked reduction in height, decreased water content, rupture of the AF, and herniation of the NP, leading to nerve impingement at the corresponding spinal segment (7). The mechanism underlying intervertebral disc degeneration has not yet been fully elucidated (8). Nevertheless, inflammation is widely accepted as the main cause of disc degeneration (2).

Among the inflammatory molecules, tumor necrosis factor-alpha (TNF-α) is thought to be the key factor associated with IVDD. The pathogenesis of IVDD involves an increase in TNF-α levels (9), and a high TNF-α level is associated with low back pain (10). The nuclear factor kappa B (NF-κB) pathway is activated when TNF-α reaches its peak during inflammation, resulting in the activation of genes related to inflammation, such as chemokines and proinflammatory cytokines like IL-1β and IL-6 (11). Besides participating in the inception and advancement of the inflammatory response, TNF-α is involved in cellular processes such as apoptosis (12). Consequently, it is possible to treat inflammatory diseases by regulating TNF expression levels. Furthermore, managing IVDD can be treated by targeting the TNF signaling pathway, which has become a feasible therapeutic choice (13).

Digoxin, a cardiac glycoside derived from foxglove, is a potent inhibitor of Na^+^/K^+^-ATPase and one of the oldest medications used for treating heart disease (14–16). Generally, digoxin is widely used in managing various cardiac diseases, including congestive heart failure and atrial fibrillation (17, 18). Several studies have shown the beneficial effects of digoxin in various inflammatory diseases, such as rheumatoid arthritis, inflammatory bowel disease, and autoimmune arthritis (19, 20). Researchers also found that digoxin treatment in mice with acute liver injury resulted in a significant decrease in proinflammatory cytokine levels, including IL-17A, IL-1β, TNF-α, and inhibited NF-κB activation (21). These studies suggest that digoxin possesses anti-inflammatory effects. Despite the anti-inflammatory effect, digoxin also demonstrated anabolic promotion in recent studies (22). However, whether digoxin suppresses TNF-α-mediated inflammation and whether digoxin has a protective effect in IVDD is still unknown.

Inflammation plays an important role in IVDD (23). The anti-inflammatory properties of digoxin have been demonstrated in different diseases (24). However, it is unclear how digoxin affects IVDD. This study explored the role of digoxin in IVDD and the associated molecular mechanisms, including the downstream proteins influenced by digoxin.

## 2 Materials and methods

### 2.1 Isolation and culture of nucleus pulposus cells

Human intervertebral disc tissue samples were obtained from spinal surgical procedures with written informed consent from the donors in accordance with ethical guidelines. The intervertebral discs were dissected, and under aseptic conditions, the NP tissue from the central part of the disc was isolated to the best possible extent. The isolated NP tissue was then minced into tissue fragments smaller than 1 mm^3^ in size. The collected NP tissue fragments were digested with 0.25% trypsin (Gibco, America) for 20 minutes, and the excess trypsin was discarded. Subsequently, the tissue fragments were enzymatically digested using type II collagenase (Solarbio, China) at a concentration of 0.2%. The tissue fragments were incubated at 37°C with 5% CO_2_ for 2–4 hours. After incubation, the digested tissue was filtered using a cell filter with a pore size of 70 μm to separate the released cells from the undigested tissue fragments. The resulting cell pellet was obtained by low-speed centrifugation (800 rpm) for five minutes. DMEM/F12 medium (Gibco, America) was used to resuspend the pellet of cells, supplemented with 10% fetal bovine serum (Gibco, America) and 1% penicillin-streptomycin (Solarbio, China). The isolated NP cells were seeded in culture flasks or dishes and maintained in a humidified incubator at 37°C with 5% CO_2_. The culture medium was replaced every two days, and subculturing was performed when the cells reached 80%–90% confluence.

Following euthanasia, mice used in the experiment were surface disinfected with 75% ethanol. Under aseptic conditions, the skin and subcutaneous tissue of the dorsal region were incised, and the paraspinal muscles were dissected to isolate the entire spine. Intervertebral disc tissue was collected, and the AF was separated under a microscope. The NP tissue was gently washed with Phosphate Buffered Saline (PBS) for five minutes. The remaining procedures for processing were similar to the previously described method for human NP cell isolation.

### 2.2 Isolation and culture of intervertebral disc tissue

Endoscopic surgical methodologies in spinal orthopedics were used to surgically extract human NP tissues from intervertebral discs. These samples were subsequently processed in a sterilized environment, which included the removal of extraneous AF and ligamentum flavum. The isolated human NP tissues were then bathed in PBS for five minutes and then transferred into a culture dish containing complete medium (DMEM/F12 medium supplemented with 10% fetal bovine serum and 1% penicillin-streptomycin), where they were cultivated for further experimentation.

Following euthanasia, the spines of mice used in the experiment were procured via previously outlined procedures. Subsequent intervertebral disc tissue isolation encompassed portions of the adjoining superior and inferior vertebral bodies, intact cartilaginous endplates, and the intervertebral disc itself. The separated murine intervertebral disc tissues were rinsed in PBS five-minute and relocated to a culture dish with complete medium, where they were cultivated for subsequent experimental use.

### 2.3 Rat needle puncture model establishment

For this experiment, eight weeks old male rats were selected and anesthetized using isoflurane inhalation. After achieving complete sedation, the rats were positioned prone on the surgical table, with their tails immobilized. The caudal vertebrae were designated as the puncture site, and a 21-gauge needle was used to penetrate the AF of the intervertebral disc. The needle was inserted parallel to the vertebral body, maintaining a depth of 5 mm. Following insertion, the needle was rotated 360° and held *in situ* for 30 seconds prior to a gradual withdrawal along the initial insertion trajectory. Subsequent to model establishment, intraperitoneal injections were administered twice weekly, containing either a low concentration of DMSO (<0.1%) or digoxin at a 50 nM concentration. Four weeks post-surgery, magnetic resonance imaging (MRI) assessments were performed under anesthesia. Upon the anticipated conclusion of the treatment regimen, rats were euthanized, and their caudal vertebrae were procured for subsequent histological examinations to assess the degree of IVDD.

### 2.4 Histological staining and analysis

The harvested intervertebral disc tissue was immersed in a 4% paraformaldehyde (Solarbio, China) fixative solution for 48 hours to preserve tissue morphology. The tissue was decalcified using a 10% EDTA solution (pH 7.2–7.4) for two weeks, with daily solution changes. Subsequently, the tissue was dehydrated through a series of graded ethanol baths, cleared with an environmentally friendly clearing agent (Solarbio, China), and embedded in paraffin wax. The paraffin-embedded tissue was then sectioned into 5 µm-thick slices using a microtome. Appropriate histological stains were then applied based on the instructions provided in the respective staining kits. Hematoxylin and eosin (H&E) staining (Solarbio, China) was used to observe the histological morphology of the intervertebral disc, while Safranin O staining (Solarbio, China) was used for visualizing cartilaginous components within the disc. Histological scoring of the H&E and Safranin O-stained samples was performed using the methodologies outlined in prior literature. The stained results were evaluated from five perspectives. Each category was assigned a score ranging from 1–3, yielding a cumulative score between 5 and 15. Higher scoring levels indicated greater degrees of degeneration (25, 26).

### 2.5 Immunohistochemical staining and analysis

Paraffin-embedded tissue sections were subjected to a thermal regimen at 65°C for an hour to facilitate the subsequent deparaffinization with an environmentally friendly clearing agent. The sections were then hydrated via a graded series of ethanol solutions. Then, sections were heated in a citrate buffer with a pH of 6.0 to retrieve antigens, after which they were allowed to cool slowly. An opaque incubation with 3% hydrogen peroxide was then conducted, followed by a 30-minute blockade with 5% Bovine Serum Albumin (BSA). Primary antibodies were diluted according to the manufacturer’s instructions, and the sections were incubated with these antibodies: COX-2 (1:100, Proteintech, China), IL-1β (1:100, Cell Signaling Technology, America), MMP-13 (1:100, Affinity, China), ADAMTS-4 (1:100, Abcam, America), Col-2 (1:100, Proteintech, China), Aggrecan (1:100, Proteintech, China), and LRP4 (1:50, Abcam, America). The sections were incubated overnight at 4°C. The following day, the sections were washed and incubated with a secondary goat anti-rabbit/mouse immunoglobulin G (IgG) antibody (ZSGB, China) at 37°C for an hour. Visualization of the antibodies was facilitated by applying 3,3’-Diaminobenzidine (DAB), followed by a counterstain with 1% hematoxylin. The images were captured and analyzed for the percentage of positive cells using the ImageJ software. For details, we measured the average gray value (staining intensity) and the percentage of positive area (stained area) of positive cells. Thus, the mean gray value is computed to evaluate the staining intensity. The procedure was taken by imageJ. The consistency of parameters is ensured when analyzing the staining intensity of each group.

### 2.6 Total RNA extraction and reverse transcription polymerase chain reaction

Total RNA from the cells was extracted using either the TRIzol reagent (Takara Bio, Japan) or a general RNA extraction kit (Fastagen, China), adhering to the manufacturer’s respective protocol, and RNA concentration was recorded. The extracted RNA was retro-transcribed into complementary DNA (cDNA) using a reverse transcription kit (Toyobo, Japan), following the specific instructions provided in the kit’s manual. This newly synthesized cDNA was then used as the template for the Polymerase Chain Reaction (PCR) to amplify specific gene sequences. A PCR system is set up with the SYBR Green-PCR Master Mix (Toyobo, Japan) serving as the dye, and the Real-time PCR reaction is executed following the instructions provided by the manufacturer. Glyceraldehyde 3-phosphate dehydrogenase (GAPDH) was used as an internal reference gene to normalize the target genes. The specificity of the PCR product was ascertained through melt curve analysis. We computed the relative levels of relative mRNA expression using the ΔΔCT method. As shown in **Table 1**, the primer sequences to the Real-time PCR experiments are listed in **Table 1**. Each experiment was replicated more than three times to ensure data reliability.

**Table 1:** Real-time PCR primers.

Target	Forward Primers,5’-3’	Reverse Primers,5’-3’
*Human*		
COX-2	TCCTTGGGTGTCAAAGGTAAA	TGGCCCTCGTTATGATCTG
iNOS	CGTGGAGACGGGAAAGAAGT	GACCCCAGGCAAGATTTGGA
IL-1β	CAACAAGTGGTGTTCTCCATGTC	ACACGCAGGACAGGTACAGA
IL-6	AGACAGCCACTCACCTCTTCA	GGCTTGTTCCTCACTACTCTC
MMP-13	ATTAAGGAGCATGGCGACTTCT	GCCCAGGAGGAAAAGCATGA
ADAMTS-4	ATGGCTATGGGCACTGTCTC	CTGGCGGTCAGCATCATAGT
Col-2	GATGGCTGCACGAAACATACC	GCCCTATGTCCACACCGAAT
Aggrecan	AAACCTGGCGTGAGAACTGT	CCACTGACACACCTCGGAAG
Bax	GAGGTCTTTTTCCGAGTGGCA	GGCAAAGTAGAAAAGGGCGAC
Bcl-2	GGGTGAACTGGGGGAGGATT	ATCTCCCGGTTGACGCTCTC
Casp3	GAGCACTGGAATGTCATCTCGCTCTG	AGACCGAGATGTCATTCCAGTGCTT
GAPDH	GCACCGTCAAGGCTGAGAAC	TGGTGAAGACGCCAGTGGA
LRP4	ACCTACCTGTTCCCCTCTTGA	GTCCTGCTCATCCGAGTCATC
*Mouse*		
COX-2	TGCTGGTGGAAAAACCTCGT	AAAACCCACTTCGCCTCCAA
iNOS	CCTGCTTTGTCGAAGTGTC	GCCAAACACCAAGCTCATGC
IL-1β	GTGTCTTTCCCGTGGACCTT	AATGGGAACGTCACACACCA
IL-6	GCCTTCTTGGGACTGATGCT	GCCATTGCACAACTCTTTTCTCA
MMP-13	TGATGATGAAACCTGGACAAGCA	GGTCCTTGGAGTGATCCAGACCTA
Col-2	CCAGATTGAGAGCATCCGCA	ACTTTCATGGCGTCCAAGGT
Aggrecan	AAACCTGGCGTGAGAACTGT	CCACTGACACACCTCGGAAG
Bax	CTGAGCTGACCTTGGAGC	GACTCCAGCCACAAAGATG
Bcl-2	TGTGGTCCATCTGACCCTCC	ACATCTCCCTGTTGACGCTCT
Casp3	AGGAGGGACGAACACGTCT	CAAAGAAGGTTGCCCCAATCT
GAPDH	CTTCACCACCATGGAGAAGGC	GACGGACACATTGGGGGTAG

### 2.7 Western blot

Human NP cells were cultivated *ex vivo* in six-well plates, with protein extraction performed at the appropriate time points. During the extraction of total cellular proteins, the culture medium was discarded from the six-well plates, followed by a gentle washing of the cells thrice using PBS. Subsequently, the cells were lysed for 30 minutes using the RIPA buffer (Solarbio, China) containing Protease Inhibitor Cocktail (NCM, China) and protein phosphatase inhibitors (NCM, China) on ice. The entire lysate was then drawn into Eppendorf tubes and further processed with a non-contact ultrasonic disintegrator (New Bioruptor Pro, Belgium). The EP tubes containing lysate were placed in a high-speed centrifuge and spun at 12,000 rpm at 4°C for 15 minutes. The supernatant was carefully aspirated into new Eppendorf tubes for storage. The nuclear protein extraction kit (Solarbio, China) instructions were followed to extract nuclear protein, allowing for the separation of nuclear and cytoplasmic proteins. The extracted proteins were initially quantified using a BCA assay kit (Solarbio, China), followed by mixing with loading buffer (EpiZyme, China) and heat denaturation before gel electrophoresis. SDS-PAGE gels were prepared based on the kDa of the proteins to be analyzed. The concentrations used in this experiment were 7.5%,10% and 12.5%. Proteins were separated in SDS-PAGE gels and transferred to PVDF membranes in equal amounts, and PVDF membranes were blocked using 5% BSA for an hour after transfer. The blocked PVDF membranes were incubated at 4°C for 12–16 hours with primary antibodies diluted according to the instructions, including COX-2 (1:1000, Proteintech, China), iNOS (1:1000, Abclonal, China), MMP-13 (1:1000, Proteintech, China), ADATMS-5 (1:1000, Abcam, America), Col-2 (1:1000, Proteintech, China), Aggrecan (1:1000, Proteintech, China), Bax (1:1000, Affinity, China), Bcl-2 (1:1000, Affinity, China), Cleaved-Caspase3 (1:1000, Affinity, China), LRP4 (1:500, Abcam, America), P-P65 (1:500, Cell Signaling Technology, America), P65 (1:1000, Affinity, China), P-AKT (1:500, Cell Signaling Technology, America), AKT (1:1000, Cell Signaling Technology, America), P-ERK1/2 (1:500, Zenbio, China), ERK1/2 (1:1000, Zenbio, China), P-IκBα (1:500, Cell Signaling Technology, America), Lamin-B1 (1:1000, Abways, China), and GAPDH (1:3000, Abways, China). The blocked PVDF membranes were washed with TBST to remove excess primary antibodies and incubated with secondary antibodies (Proteintech, China) for one hour, followed by image acquisition using a chemiluminescence imaging analyzer (Tanon5200, China). Image analysis was conducted using ImageJ software.

### 2.8 Immunofluorescence staining

Cultures of human NP cells were performed in 24-well plates to achieve the desired density and fixed with 4% paraformaldehyde. After fixation, the cells were permeabilized for 10 minutes with 0.2% Triton X-100 and then blocked for 30 minutes with 1% BSA to impede nonspecific antibody binding. Thereafter, the cells were incubated overnight at 4°C with specifically diluted primary antibodies as per the instructions provided. The primary antibodies involved in this experiment included COX-2(1:200, Proteintech, China), MMP-13(1:200, Affinity, China), and C-Caspase3(1:200, Affinity, China). On the following day, the cells were rinsed three times for five minutes each with PBS containing Tween-20 to remove excess primary antibodies. Then, the cells were incubated for an hour at room temperature with a fluorescently labeled goat anti-rabbit secondary antibody (1:200 ZSGB-BIO, China). The cells are then washed three times with PBS and treated with an appropriate amount of DAPI-containing anti-quenching agent (Solarbio, China) to ensure complete and uniform coverage of the cell surface. Finally, fluorescence images were captured using an inverted fluorescence microscope and measured using the software. In order to compare the fluorescence intensity, we measured the fluorescent area ImageJ and integrated the density of each image. The mean fluorescence intensity of each image was then analyzed while ensuring consistent parameters.

### 2.9 TUNEL staining

Human NP cells were inoculated into 24-well plates and stimulated with TNF-α for 48 hours to induce apoptosis. The treatment group was pre-treated with 50 nM of digoxin. Following the manufacturer’s instructions, cells were stained using the TUNEL assay kit (ELabScience Biotech, China), and images were captured using an inverted fluorescence microscope.

### 2.10 3-(4,5-dimethylthiazol-2-yl)-2,5-diphenyltetrazolium bromide assay (MTT assay)

Human NP cells are cultivated *in vitro* in a 96-well plate, with each well incubated with 100 µL of complete culture medium. After 24 hours, the cells were treated with digoxin at varying concentrations of 10, 20, 30, 50, and 100 nM and concurrently incubated for an additional 24 hours. Subsequently, MTT assays were performed according to the instructions provided with the MTT kit (Solarbio, China), culminating in the measurement of absorbance at a wavelength of 570 nm. The cell viability was represented as the percentage of the average cell survival rate in the treatment groups relative to that in the control group.

### 2.11 Alcian Blue staining

Human NP cells, isolated from original tissue, were uniformly distributed into a six-well plate, with each well containing 2 mL of complete medium for optimal growth conditions. The experimental group of cells was treated with 50 nM digoxin. Subsequently, on the 3^rd^, 7^th^, and 14^th^ days post-inoculation, these cells were stained with the Alcian Blue Stain Kit For Cell (Solarbio, China) according to the manufacturer’s detailed instructions. To compare the relative staining level of Alcian Blue, we measured the fluorescent area and integrated the density of each image using the ImageJ software. The relative staining level of each image was analyzed while ensuring consistent parameters.

### 2.12 Transfection

The NP cells derived from humans were cultured in complete media until they attained an approximate confluence of 40%–50%, after which the media was promptly discarded, and the cells were rinsed with PBS. Subsequently, siRNA (Ribo, China) and transfection reagent (Ribo, China) were meticulously combined in accordance with the manufacturer’s guidelines. This synergistic mixture was then incorporated into an antibiotic-free complete media environment to facilitate cell cultivation. A series of downstream experiments were performed after 48 hours. The effectiveness of the gene knockdown was subsequently verified using Western blot analysis. The protocol for transfection with overexpression plasmids was similar to the previously detailed process.

### 2.13 Ethics statement

This research was approved by the Medical Ethics Committee at Qilu Hospital of Shandong University. During the study, 31 patients—17 women and 14 men, aged 18–65 years—voluntarily participated and gave their informed consent. All participants underwent lumbar disc excision surgery at Qilu Hospital of Shandong University, and the collection of intervertebral disc tissue adhered to medical standards. Prior to surgery, MRI scans of the patients were obtained, and the collected human NP tissue samples were graded according to the Pfirrmann classification system based on the intensity of T2-weighted (T2WI) signals in the intervertebral discs. These samples were then used for further experimental study. The details of the patients of the sample provided in **Table 2**. All animal experiments involved in this study were conducted following the International Guiding Principles for Animal research, were approved by the Animal Experimentation Center of Shandong University and met the welfare requirements for experimental animals.

**Table 2:** Summary of clinical and demographic features of patients.

Subject Number	Gender	Age	Level	Pfirrmann Grade
1	Female	29	L4/5	II
2	Male	18	L4/5	II
3	Female	31	L5/S1	II
4	Female	27	L4/5	II
5	Male	22	L5/S1	II
6	Female	28	L5/S1	II
7	Male	34	L4/5	II
8	Female	37	L5/S1	II
9	Female	25	L5/S1	II
10	Male	51	L4/5	III
11	Female	47	L4/5	III
12	Male	43	L5/S1	III
13	Female	36	L5/S1	III
14	Male	41	L5/S1	III
15	Female	40	L4/5	III
16	Male	53	L5/S1	III
17	Female	55	L4/5	IV
18	Female	58	L5/S1	IV
19	Female	49	L4/5	IV
20	Male	57	L5/S1	IV
21	Male	63	L4/5	IV
22	Male	60	L4/5	IV
23	Female	55	L5/S1	IV
24	Male	58	L5/S1	IV
25	Female	49	L5/S1	V
26	Male	65	L4/5	V
27	Male	62	L5/S1	V
28	Female	57	L4/5	V
29	Male	60	L4/5	V
30	Female	62	L5/S1	V
31	Female	59	L4/5	V

### 2.14 Statistical analysis

The present investigation used a blind method to gather all experimental data. Statistical analysis was performed in GraphPad Prism 9. T-tests were used to compare the means of two groups, while the analysis of variance (ANOVA) was used for comparisons involving more than two groups. Depending on the specific research context, ANOVA could be either one-way (one independent variable) or two-way (two independent variables), with Tukey’s HSD *post hoc* test conducted using SPSS 22.0 software following the variance analysis. A p-value of less than 0.05 was considered statistically significant. Each value was expressed as the mean ± Standard Error of the Mean (SEM).

## 3 Results

### 3.1 Digoxin inhibited TNF-α-induced inflammation in the disc

**Figure 1:**
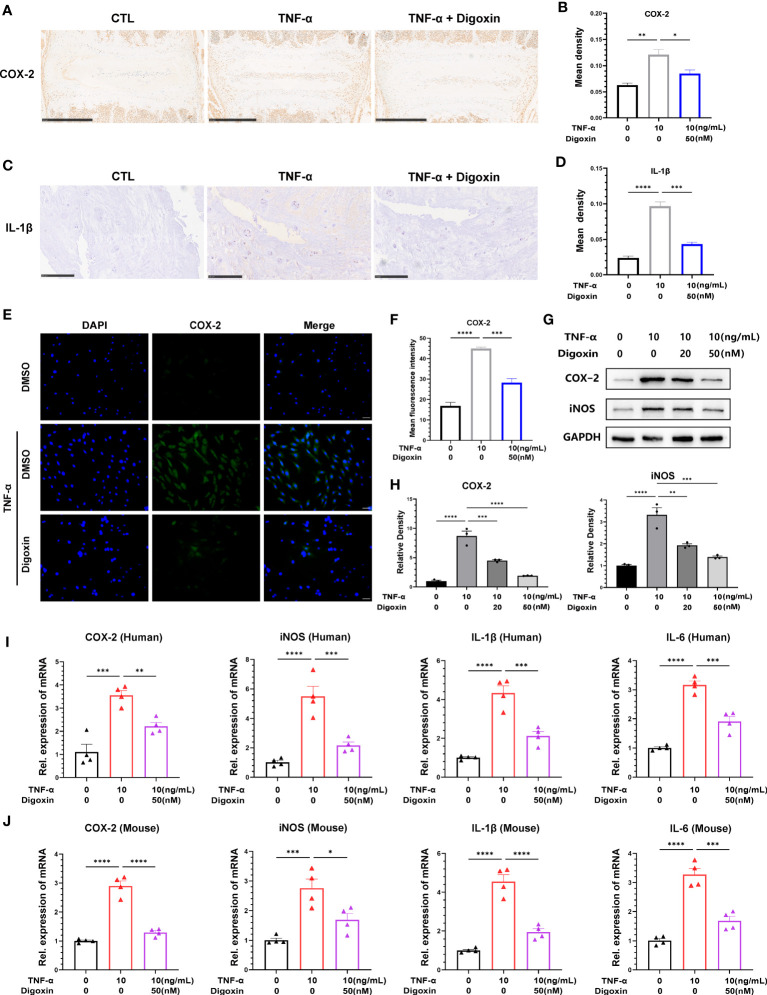
Digoxin inhibited TNF-α-induced inflammation in intervertebral disc. **(A)** Immunohistochemical staining of COX-2 in mouse disc tissues. The brown signal indicates positive. Scale bar = 500 µm. **(B)** The mean density of brown signal based on panel A; six groups of mice intervertebral disc tissues were used for observation. **(C)** Immunohistochemical staining of IL-1β in human disc tissues. The brown signal indicates positive. Scale bar = 250 µm. **(D)** The mean density of the brown signal based on panel **(C)**, six groups of human nucleus pulposus tissues were used for observation. **(E)** Immunofluorescence staining of COX-2 in human nucleus pulposus cells. Scale bar = 100 µm. **(F)** The mean density of green signal based on panel **(E)**. **(G)** The impact of different concentrations of digoxin on the protein expression of COX-2 and iNOS was assessed in human nucleus pulposus cells treated with 10 ng/mL TNF-α for 48 hours using Western blot analysis. **(H)** Relative density of bands based on Western blot. **(I, J)** The effect of digoxin on the transcriptional levels of COX-2, iNOS, IL-1β, and IL-6 was evaluated in human/mouse nucleus pulposus cells treated with 10 ng/mL TNF-α for 24 hours using Real-time PCR assay. The statistical analysis used ordinary one-way analysis of variance (ANOVA) to assess the differences between groups, followed by Tukey’s multiple comparisons test as a *post hoc* analysis. The values shown above represent the mean ± standard error of the mean (SEM) * P ≤ 0.05, ** P ≤ 0.01, *** P ≤ 0.001, **** P ≤ 0.0001 versus the comparison group.

To determine whether digoxin has an anti-TNF-α-induced inflammatory effect in IVDD, we isolated mouse intervertebral disc tissue for *ex-vivo* culture and performed immunohistochemical staining for cyclooxygenase-2 (COX-2). We found that digoxin downregulated TNF-α-induced COX-2 expression compared to the control group (**Figures 1A, B**). Furthermore, immunohistochemical staining of cultured human NP tissue indicated that digoxin inhibited IL-1β expression *in vitro* (**Figures 1C, D**). Additionally, the immunofluorescence staining of COX-2 demonstrated that digoxin significantly suppressed the TNF-α-induced inflammatory response in primary human NP cells (**Figures 1E, F**). To further confirm this finding at the protein level, we isolated human nucleus NP for *in vitro* culture. As shown in **Figures 1G, H**, Western blot results showed upregulation in the protein expression of COX-2 and inducible nitric oxide synthase (iNOS) in the presence of TNF-α, while additional use of digoxin decreased their expression. Real-time PCR analysis on both human and mouse NP cells was conducted to determine transcriptional levels of proinflammatory cytokines. As **Figures 1I, J** indicate, digoxin significantly reduced TNF-α-induced cytokines mRNA expression, including COX-2, iNOS, IL-1β, and IL-6.

### 3.2 Digoxin protects against intervertebral disc from matrix destruction

**Figure 2:**
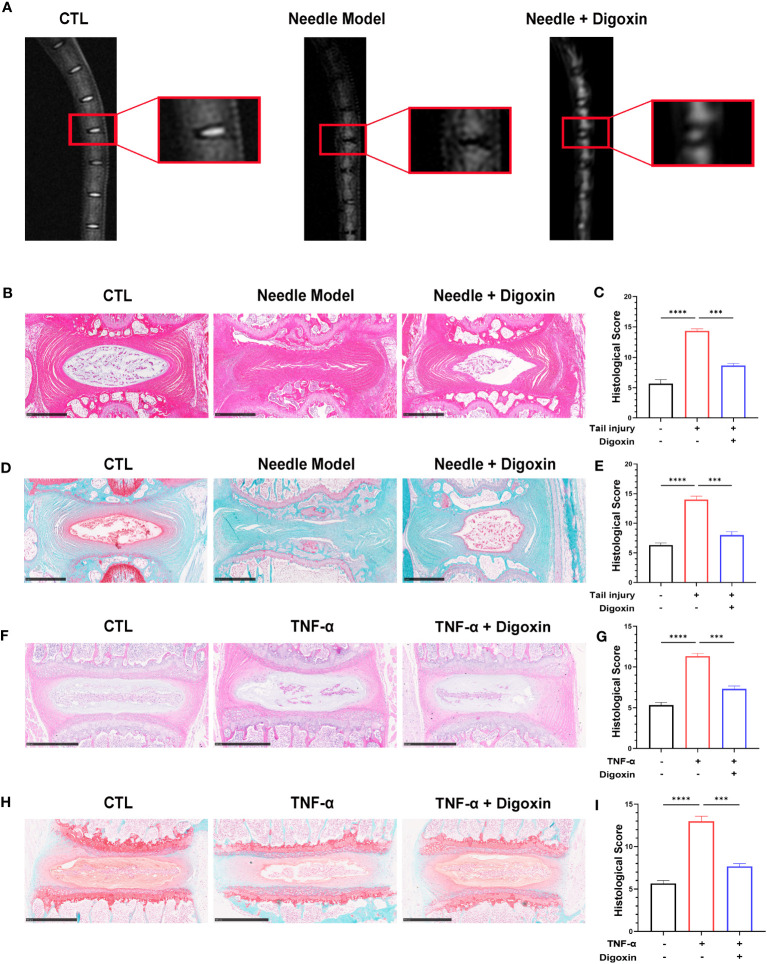
Digoxin alleviated intervertebral disc degeneration in the murine model. **(A)** T2WI MRI images of rat intervertebral discs after establishing the puncture model—each group comprised six adult rats aged eight weeks old. **(B)** Hematoxylin and Eosin (H&E) staining of rat intervertebral discs puncture model. Scale bar = 1 mm. **(C)** Histological score assessment based on H&E staining. **(D)** Safranin O and Fast Green staining of rat intervertebral discs puncture model. Scale bar = 1 mm. **(E)** Histological score assessment based on Safranin O and Fast Green staining. **(F, G)** H&E staining was conducted on mouse intervertebral disc tissue with or without TNF-α *ex vitro*, and histological scoring was performed to assess the tissue changes. Scale bar = 500 µm. **(H, I)** Safranin O and Fast Green staining was performed on mouse intervertebral disc tissue with or without TNF-α *ex vitro*, and histological scoring was conducted to evaluate the tissue changes. Scale bar = 500 µm. The intervertebral disc tissues of six groups of rats were used for observation. The statistical analysis employed ordinary one-way ANOVA to assess the differences between groups, followed by Tukey’s multiple comparisons test as a *post hoc* analysis. The values shown above represent the mean ± SEM ***p < 0.001 and ****p < 0.001 versus the comparison group.

We developed a rat puncture model to further investigate the protective effects of digoxin on IVDD. T2-weighted Magnetic Resonance Imaging (T2WI MRI) showed that the disc signal intensity and intervertebral space height of the digoxin-treated group rats were higher than those of the puncture group rats (**Figure 2A**). Subsequently, tissue samples were collected for histological staining and scoring. As shown in **Figures 2B–E**, intervertebral discs treated with digoxin displayed better histological morphology in H&E staining. Additionally, Safranin O/Fast Green staining demonstrated a reduction in loss of cartilaginous components in the intervertebral disc tissue after treatment with digoxin compared to the puncture group, which is considered a morphological manifestation of IVDD. Additionally, we also performed histological staining and scoring on mouse intervertebral discs cultured *ex vivo*. The results of H&E staining analysis indicated a significant reduction in histological scores for intervertebral discs treated with digoxin compared to the TNF-α stimulated group (**Figures 2F, G**). Furthermore, Safranin O/Fast Green staining showed that digoxin reduced the decrease in cartilage content induced by TNF-α in mouse intervertebral discs (**Figures 2H, I**).

**Figure 3:**
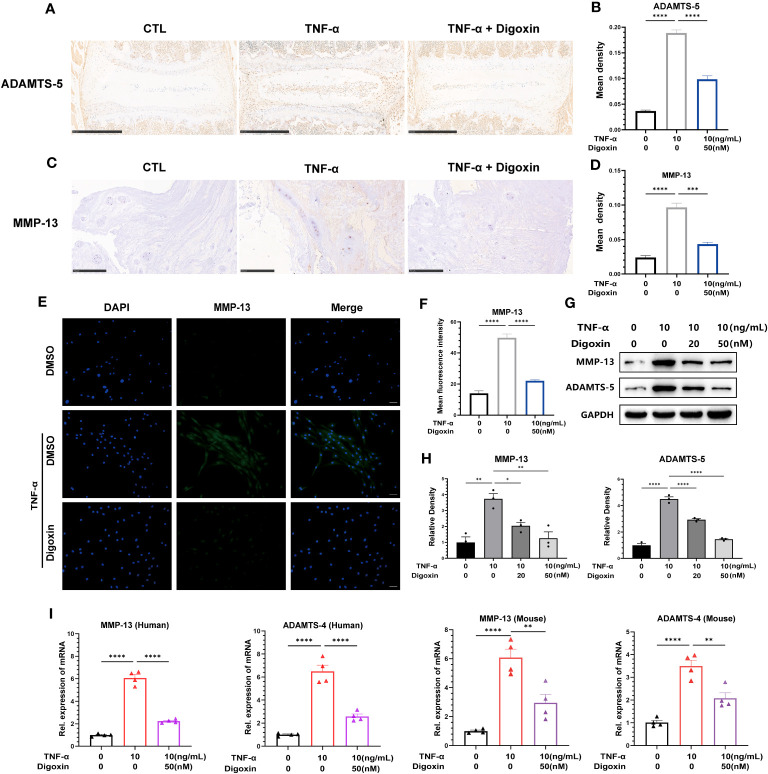
Digoxin inhibits TNF-α-mediated metalloproteinase expression in intervertebral discs. **(A)** Immunohistochemical staining analysis of ADAMTS-5 was conducted on *ex-vitro* mouse intervertebral disc tissues treated with 10 ng/mL TNF-α, with or without 50 nM digoxin. The brown signal indicates positive. Scale bar = 500 µm. **(B)** The mean density of brown signal based on panel A; six groups of mice intervertebral disc tissues were utilized for observation. **(C)** Immunohistochemical staining analysis of MMP-13 in human nucleus pulposus tissues cultured *ex vitro* under conditions of TNF-α treatment, with or without 50 nM Digoxin. The brown signal indicates positive. Scale bar = 250 µm. **(D)** The mean density of the brown signal based on panel C, six groups of human nucleus pulposus tissues were used for observation. **(E)** Immunofluorescence staining of MMP-13 in human nucleus pulposus cells in the presence or absence of 50 nM digoxin with or without TNF-α treatment. The green signal indicates positive. Scale bar = 100 µm. **(F)** The mean fluorescence intensity of green signal based on **(E)**. **(G)** Western blot analysis was performed to examine the expression of MMP-13 and ADAMTS-5 proteins in human nucleus pulposus cells treated with different concentrations of digoxin in the presence or absence of 10 ng/mL TNF-α. **(H)** Relative density of bands based on Western blot. **(I)** Real-time PCR assay was employed to assess the changes in transcriptional levels of MMP-13 and ADAMTS-4 in human and mouse nucleus pulposus cells, with or without 10 ng/mL TNF-α, and in the presence or absence of 50 nM digoxin. Ordinary one-way ANOVA was used to assess the differences between groups, followed by Tukey’s multiple comparisons test as a *post hoc* analysis. The values shown above represent the mean ± SEM * P ≤ 0.05, ** P ≤ 0.01, *** P ≤ 0.001, **** P ≤ 0.0001 versus the comparison group.

The extracellular matrix (ECM) of the NP is mainly maintained by type II collagen (Col-2) and Aggrecan in a healthy state, and ECM degradation is crucial for disc degeneration. To explore the effect of digoxin on the regulation of ECM degradation in intervertebral discs, we performed *ex vitro*-cultured mouse intervertebral discs and human NP tissues. As shown in **Figures 3A, B**, digoxin downregulated the TNF-α-induced increase of A Disintegrin And Metalloproteinase with ThromboSpondin motif 5 (ADAMTS-5) in the mouse intervertebral disc. **Figures 3C, D** demonstrated that digoxin inhibited the TNF-α-mediated expression of matrix metallopeptidase 13 (MMP-13) in human disc tissue. The cell immunofluorescence staining for MMP-13 in primary HNPCs also showed that digoxin suppressed TNF-α-mediated acceleration of ECM degradation metalloproteinase MMP-13 (**Figures 3E, F**). A Western blot assay was performed to investigate the involved molecular mechanism further. As shown in **Figures 3G, H**, TNF-α increased MMP-13 and ADAMTS-5 levels, and additional use of digoxin showed decreased expression of metalloproteinases. **Figure 3I** demonstrated that digoxin significantly reduced TNF-α-induced the expression of ECM degradation metalloproteinases, including MMP-13 and ADAMTS-4, in both mouse and human NP cells. These findings suggest digoxin reduced TNF-α-mediated matrix metalloproteinase expression, leading to decreased matrix destruction.

**Figure 4:**
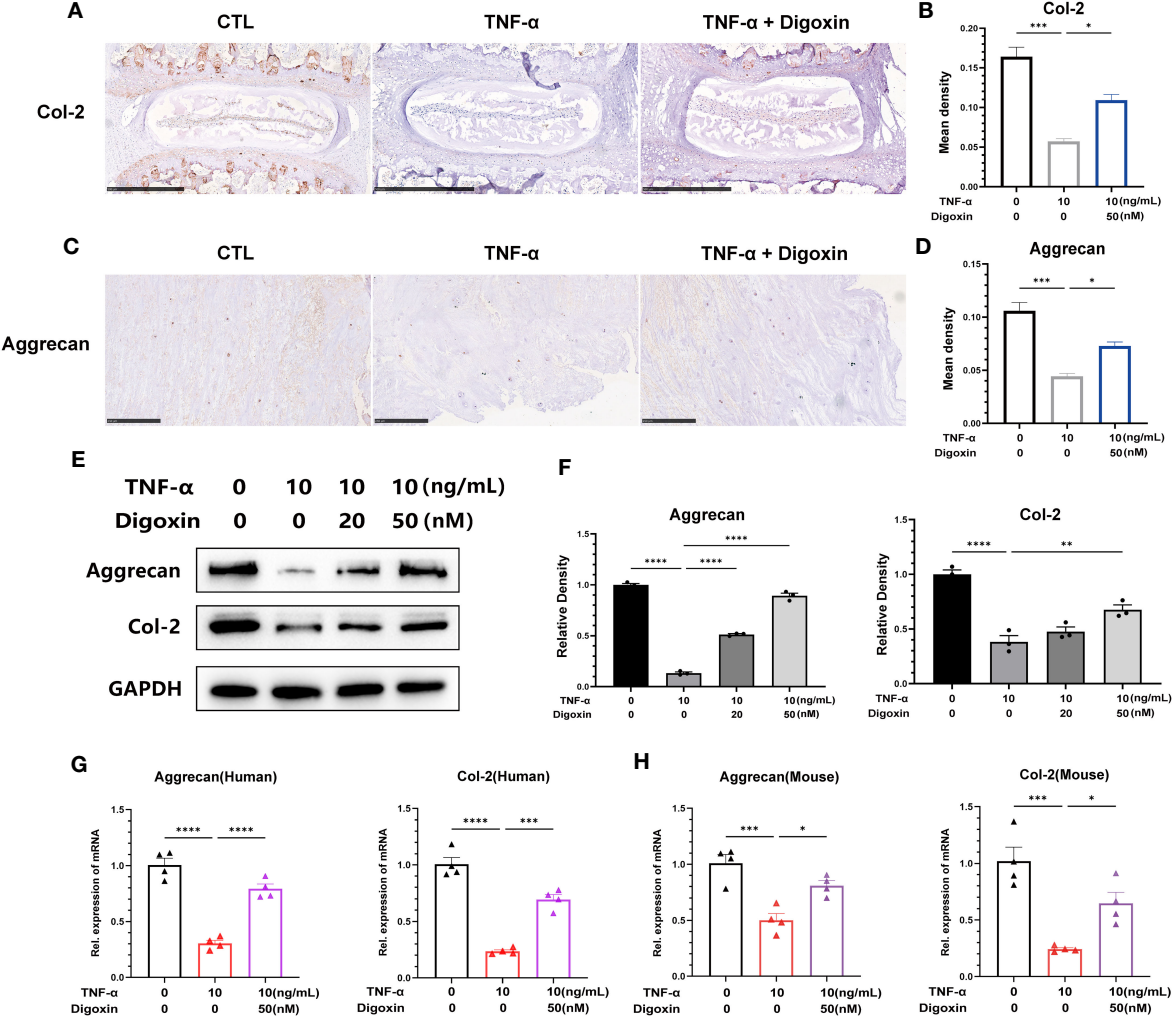
Digoxin attenuates TNF-α-mediated extracellular matrix loss in discs. **(A)** Immunohistochemical analysis of Col-2 in mouse disc tissue in the presence or absence of 10 ng/mL TNF-α, with or without 50 nM digoxin. The brown signal indicates positive. Scale bar = 500 µm. **(B)** The mean density of brown signal based on panel A; six groups of mice intervertebral disc tissues were used for observation. **(C)** Immunohistochemical analysis of Aggrecan in human disc tissue cultured in the presence or absence of 10 ng/mL TNF-α, with or without 50 nM digoxin. The brown signal indicates positive. Scale bar = 250 µm. **(D)** The mean density of the brown signal based on panel C, six groups of human nucleus pulposus tissues were used for observation. **(E)** Human nucleus pulposus cells were cultured *in vitro* and treated with or without 10 ng/mL TNF-α, along with 20 nM or 50 nM digoxin or without digoxin. Western blot analysis was employed to assess the protein levels of Col-2 and Aggrecan. **(F)** Relative density of bands based on Western blot. **(G, H)** Human or mouse nucleus pulposus cells were cultured *in vitro* in the presence or absence of 10 ng/mL TNF-α, with or without 50 nM digoxin. Real-time PCR analysis was performed to analyze the relative transcriptional levels of Col-2 and Aggrecan. Ordinary one-way ANOVA was used to assess the differences between groups, followed by Tukey’s multiple comparisons test as a *post hoc* analysis. The values shown above represent the mean ± SEM * P ≤ 0.05, ** P ≤ 0.01, *** P ≤ 0.001, **** P ≤ 0.0001 versus the comparison group.

To confirm the defensive impact of digoxin on ECM degradation mediated by TNF-α, we directly measured the expression levels of Col-2 and Aggrecan. As illustrated in **Figures 4A, B**, *ex-vivo* culture of murine discs revealed significant inhibition of Col-2 loss by digoxin. Additionally, in human disc tissue, TNF-α dramatically caused Aggrecan loss, but the process was effectively reversed by digoxin (**Figures 4C, D**). To address the involved pathway, primary human NP cells were cultured. **Figures 4E, F** revealed that digoxin significantly reversed TNF-α-mediated Col-2 and Aggrecan loss *in vitro* by Western blot assay. Accordingly, Real-time PCR was performed in human and mouse NP cells to determine the transcriptional level change. As revealed in **Figures 4G, H**, digoxin effectively reversed the decreased expression of Col-2 and Aggrecan after TNF-α stimulation.

Collectively, digoxin exerted its matrix protective effect by inhibiting destructive metalloproteinase and rescuing TNF-α-mediated matrix loss.

### 3.3 Digoxin inhibits TNF-α-induced nucleus pulposus cell apoptosis

**Figure 5:**
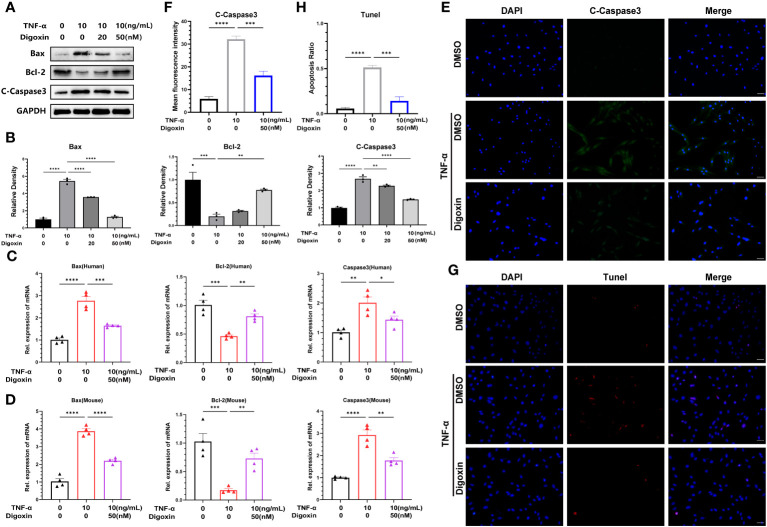
Digoxin inhibits TNF-α-induced apoptosis of nucleus pulposus cells. **(A, B)** The changes in Bax, Bcl-2, and Cleaved-Caspase3 proteins were assessed by Western blot analysis in human nucleus pulposus cells cultured *in vitro* and treated with 10 ng/mL TNF-α, with various concentrations of Digoxin. **(C, D)** Real-time PCR was performed to evaluate the relative mRNA levels of Bax, Bcl-2, and Caspase3 in human or mouse nucleus pulposus cells in the presence or absence of 10 ng/mL TNF-α, with or without 50 nM Digoxin. **(E, F)** Immunofluorescence analysis was conducted to examine the protein expression of Cleaved-Caspase3 in human nucleus pulposus cells stimulated with TNF-α, with or without 50 nM Digoxin. Scale bar = 100 µm. **(G, H)** TUNEL staining was used to analyze the nuclear DNA fragmentation in human nucleus pulposus cells treated with 50 nM digoxin following stimulation with 10 ng/mL TNF-α. Scale bar = 100 µm. Ordinary one-way ANOVA was used to assess the differences between groups, followed by Tukey’s multiple comparisons test as a *post hoc* analysis. The values shown above represent the mean ± SEM * P ≤ 0.05, ** P ≤ 0.01, *** P ≤ 0.001, **** P ≤ 0.0001 versus the comparison group.

It is thought that NP cells apoptosis plays a key role in disc degeneration. We hypothesized whether digoxin could counteract TNF-α-induced apoptosis of NP cells. To address this issue, we examined the apoptosis-related proteins in HNPCs with or without digoxin in the presence or absence of TNF-α. As shown in **Figures 5A, B**, digoxin rescued the TNF-α-mediated increased Bcl-2-associated X protein (Bax) and Cleaved-Caspase3 (C-Caspase3) levels and restored the expression of B-cell lymphoma 2 (Bcl-2) in the presence of TNF-α. Real-time PCR results for human and mouse NP cells showed that Bcl-2 mRNA levels increased with the additional use of digoxin compared to the TNF-α-stimulated group. However, Bax and Caspase3 transcriptional levels decreased with digoxin treatment (**Figures 5C, D**). Immunofluorescence staining for C-Caspase3 showed that HNPCs treated with digoxin had lower fluorescence intensity compared to those stimulated with TNF-α (**Figures 5E, F**). TUNEL (terminal deoxynucleotidyl transferase nick end labeling) is a frequently used method to detect DNA fragments, a hallmark of apoptotic cell death. As shown in **Figures 5G, H**, TUNEL staining of HNPCs demonstrated that digoxin reduced TNF-α-induced DNA fragmentation and inhibited HNPCs apoptosis.

### 3.4 Digoxin suppressed TNF-α-mediated inflammation via NF-κB signaling

**Figure 6:**
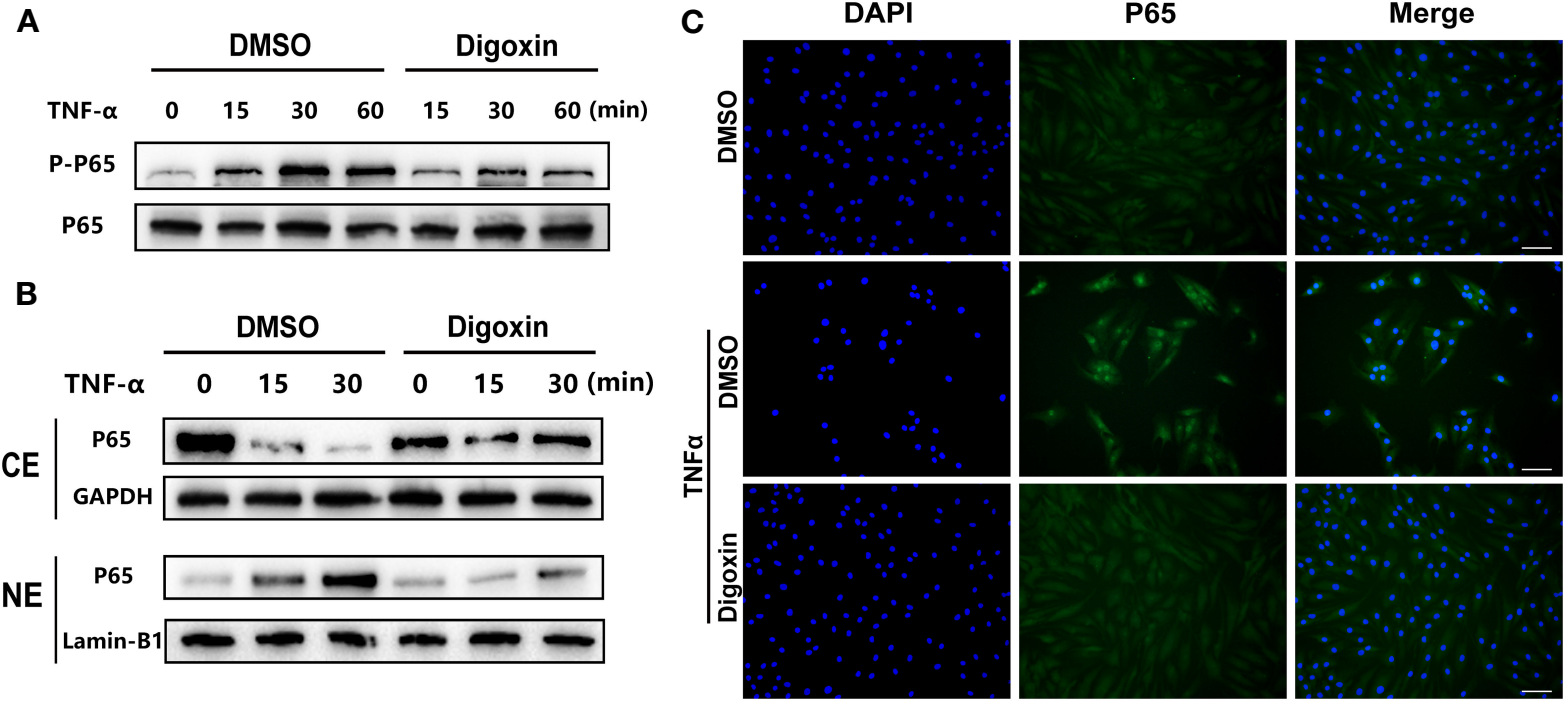
Digoxin suppressed TNF-α/NF-κB signaling pathway. **(A)** Human nucleus pulposus cells were treated with TNF-α in the presence or absence of 50 nM Digoxin. The total expression of p65 and phosphorylated p65 at various time points were analyzed by Western blot analysis. **(B)** Human nucleus pulposus cells were treated with TNF-α in the presence or absence of 50 nM digoxin. Nuclear and cytoplasmic protein was collected separately and followed by Western blot analysis for p65 expression. **(C)** Immunofluorescence staining of p65 protein in human nucleus pulposus cells after 30 minutes of TNF-α stimulation, in the presence or absence of 50 nM Digoxin. Scale bar = 100 µm.

NF-κB signaling plays a critical role in the pathogenesis of IVDD by triggering inflammation, ECM degradation, and cell death. Owing to the ability of digoxin to counteract TNF-α-induced disc degeneration, we then sort to determine the underlying mechanism. As shown in **Figure 6A**, TNF-α strongly activated the phosphorylation of p65 and effectively reduced TNF-α-induced p65 phosphorylation. Furthermore, cytoplasmic and nuclear proteins were extracted from HNPCs for Western blotting. The results showed that TNF-α stimulation caused a time-dependent decrease in cytoplasmic p65 expression and an increase in nuclear p65 expression, while digoxin treatment significantly attenuated this trend (**Figure 6B**). Immunofluorescence staining showed that digoxin significantly inhibited TNF-α-induced nuclear translocation of p65 in HNPCs, further confirming the effect of digoxin on TNF-α-mediated activation of the NF-κB pathway (**Figure 6C**).

### 3.5 Digoxin promotes anabolism via AKT and ERK1/2 pathways

**Figure 7:**
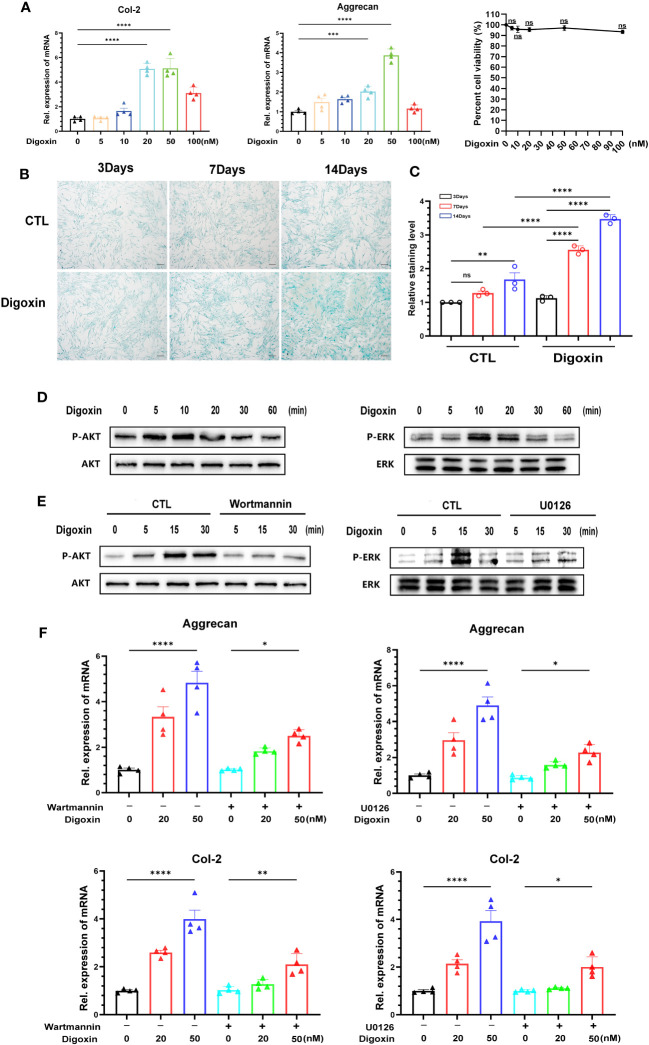
Digoxin promotes anabolism through AKT and ERK1/2 pathways. **(A)** Transcriptional levels of Col-2 and Aggrecan were assessed by Real-time PCR assay after 24 hours of treatment with different concentrations of digoxin in human nucleus pulposus cells. The impact of different digoxin concentrations on cell viability was analyzed using the MTT assay. **(B)** Alcian Blue staining was performed to analyze proteoglycan expression in human discs after treatment with 50 nM digoxin at days 3, 7, and 14. Scale bar = 200 µm. **(C)** The mean density of the blue signal is based on Alcian Blue staining. **(D)** Western blot analysis was conducted to evaluate the phosphorylation and total levels of AKT and ERK1/2 in human nucleus pulposus cells in the presence of 50 nM digoxin at various time points. **(E)** Phosphorylation and total levels of AKT and ERK1/2 in human nucleus pulposus cells at various time points in the presence of 50 nM digoxin with or without Wortmannin or U0126. **(F)** Transcriptional levels of Aggrecan and Col-2 in human nucleus pulposus cells treated with 50 nM digoxin, with or without Wortmannin or U0126. Ordinary one-way ANOVA was used to assess the differences between groups, followed by Tukey’s multiple comparisons test as a *post hoc* analysis. The values shown above represent the mean ± SEM * P ≤ 0.05, ** P ≤ 0.01, *** P ≤ 0.001, **** P ≤ 0.0001 versus the comparison group. ns, no significance.

To determine whether digoxin can directly promote the anabolism in discs, we used culture media containing different concentrations of digoxin to culture primary HNPCs. As shown in **Figure 7A**, treatment with 20 nM and 50 nM digoxin significantly upregulated the transcriptional levels of Col-2 and Aggrecan. The MTT assay results demonstrated that the use of digoxin did not affect the viability of NP cells. Subsequently, HNPCs were cultured in a medium supplemented with 50 nM digoxin, and Alcian Blue staining was performed on days 3, 7, and 14. Following the addition of 50 nM digoxin, the expression levels of proteoglycans in NPCs were significantly upregulated on the 3^rd^, 7^th^, and 14^th^ days. Furthermore, these expression levels were higher compared to the control group, indicating a significant increase in proteoglycan expression induced by digoxin treatment (**Figures 7B, C**). The ERK1/2 and AKT pathways play crucial roles in regulating ECM synthesis (27, 28). To investigate whether the activation of the ERK1/2 and AKT pathways is involved in digoxin-mediated anabolism, we stimulated HNPCs with 50 nM digoxin and extracted total proteins at different time points, followed by Western blot analysis. The results depicted in **Figure 7D** demonstrated that AKT and ERK1/2 were phosphorylated by the addition of digoxin. To further confirm this finding, specific pathway inhibitors (U0126, Wortmannin) were added. As illustrated in **Figure 7E**, the phosphorylation of ERK1/2 and AKT decreased. Moreover, to confirm whether the pro-anabolism of digoxin depends on the activation of the ERK1/2 and AKT pathways, we pre-treated HNPCs with U0126 or Wortmannin for one hour, followed by treatment with 50 nM digoxin for 24 hours. The Real-time PCR results showed a decreased expression of Col-2 and Aggrecan at transcriptional levels, indicating that digoxin promotes ECM anabolism in HNPCs through the ERK1/2 and AKT pathways **(** **Figure 7F** **)**.

### 3.6 Digoxin protects against intervertebral disc degeneration target LRP4

**Figure 8:**
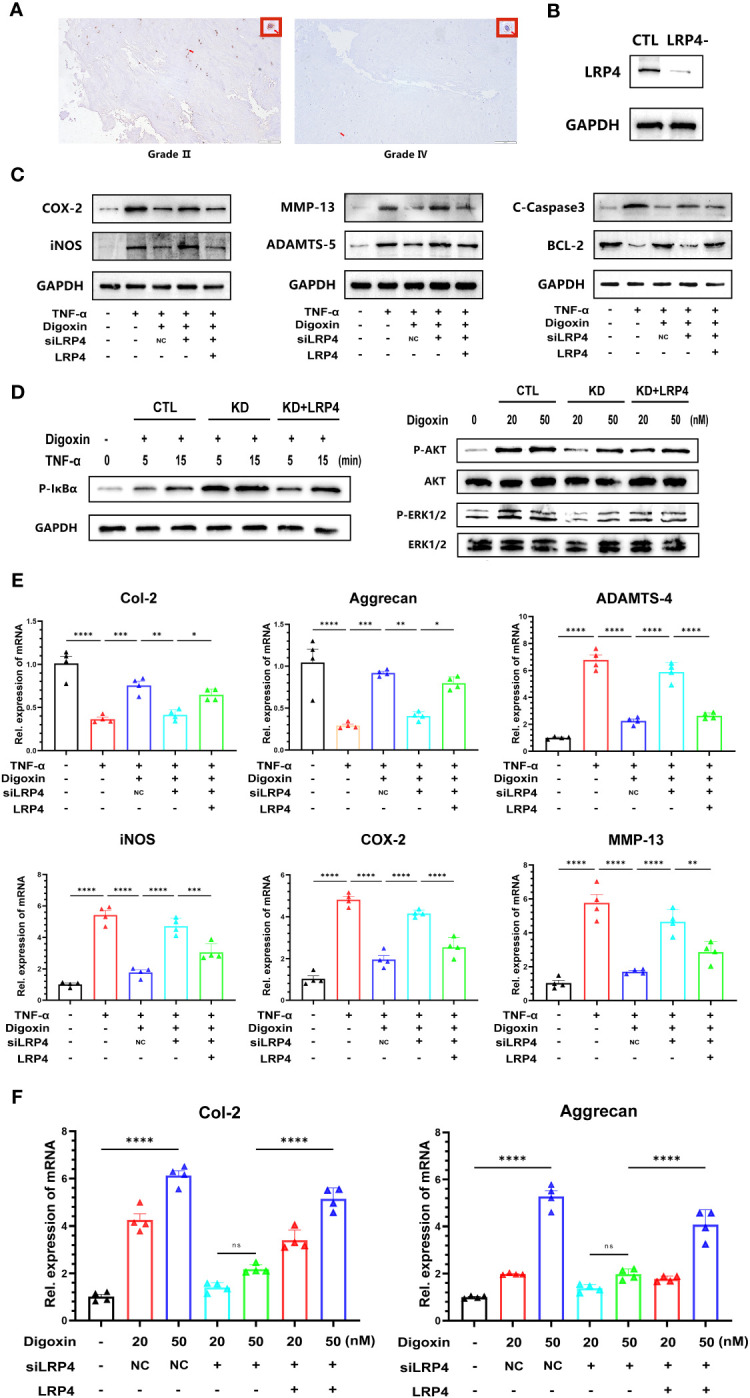
Digoxin protects against intervertebral disc degeneration via targeting LRP4. **(A)** Immunohistochemistry staining of LRP4 in human disc tissue. The first lane was from a Pfirrmann grade II disc, while the second lane was from a Pfirrmann grade IV disc. Scale bar = 200 µm. **(B)** Western blot analysis was conducted to examine the expression of LRP4 protein in primary human nucleus pulposus cells following siRNA transfection. **(C)** Western blot analysis was performed to evaluate the protein levels of COX-2, iNOS, MMP-13, ADAMTS-5, Bcl-2, and Cleaved Caspase-3 in human nucleus pulposus cells. LRP4 knockdown, KD, Negative control, NC. **(D)** Western blot for phosphorylated and total IκBα, phosphorylated and total AKT, and phosphorylated and total ERK1/2 after LRP4 knockdown and backup. GAPDH was examined as an internal control. **(E)** Real-time PCR assay was used to detect transcriptional levels of Col-2, Aggrecan, ADAMTS-4, iNOS, COX-2, and MMP-13 in primary human nucleus pulposus cells with LRP4 knockdown or LRP4 expression recovery, with or without treatment of 10 ng/mL TNF-α and with or without 50 nM digoxin. **(F)** Real-time PCR assay was used to analyze transcriptional levels of Col-2 and Aggrecan in human nucleus pulposus cells with LRP4 knockdown or LRP4 backup after treatment with different concentrations of digoxin. Ordinary one-way ANOVA was used to assess the differences between groups, followed by Tukey’s multiple comparisons test as a *post hoc* analysis. The values shown above represent the mean ± SEM * P ≤ 0.05, ** P ≤ 0.01, *** P ≤ 0.001, **** P ≤ 0.0001 versus the comparison group. ns, no significance.

Digoxin is a Na^+^/K^+^-ATPase inhibitor widely used in clinical practice, and its inhibitory effect on Na^+^/K^+^-ATPase is well-recognized. However, surprisingly, we have found that the protective effect exerted by digoxin seems to be unrelated to the inhibition of Na^+^/K^+^-ATPase (**Supplementary Figure 1A**). Low-density lipoprotein receptor-related protein 4 (LRP4) is a transmembrane protein involved in various cellular processes, including cell adhesion, migration, and signal transduction (29, 30). Recent studies have indicated that LRP4 is a novel target involved in digoxin-mediated regulation of cartilage metabolism (22). The results from **Figure 8A** and **Supplementary Figure 1B** also demonstrate a decrease in LRP4 expression disc with increasing grades of IVDD. However, The results of the Western blot analysis showed that digoxin attenuated the degradation of LRP4 in the rat needle model (**Supplementary Figure 1C**). To investigate whether the protective effect of digoxin against disc degeneration depended on LRP4 protein, we cultured primary human NP and transfected siRNA to knock down LRP4. As shown in **Figure 8B**, HNPCs exhibited a significant LRP4 expression reduction. Then, the transfected cells were stimulated with TNF-α in the absence or presence of 50 nM digoxin. After 48 hours, total proteins were extracted for Western blot analysis. **Figure 8C** demonstrates that in the presence of digoxin, the siLRP4 group showed elevated expression of COX-2, iNOS, MMP-13, ADAMTS-5, and C-Caspase3, while the protein expression of Bcl-2 decreased. However, after restoring LRP4, this trend was reversed. Next, to determine whether the anti-inflammatory and ECM synthesis-promoting effects of digoxin depended on LRP4 protein, we used Western blotting for the signaling test. The Western blot results showed that LRP4 knockdown increased the phosphorylation of IκBa, while restoration of LRP4 expression rescued this process (**Figure 8D**). Meanwhile, inhibition of digoxin-mediated activation of ERK1/2 and AKT signaling was observed in LRP4-knockdown HNPCs. However, this inhibitory effect was attenuated when LRP4 was restored (**Figure 8D**). Next, we assessed the mRNA levels of inflammation and ECM metabolism-related genes in transfected HNPCs. Consistent with the protein level analysis, the Real-time PCR results revealed that LRP4 knockdown increased the mRNA levels of COX-2, iNOS, MMP-13, and ADAMTS-4, and a decrease in mRNA levels of Col-2 and Aggrecan in TNF-α-stimulated HNPCs. This trend was not reversed by prior treatment with digoxin. However, upon restoration of LRP4 expression, the digoxin-treated group exhibited a decrease in mRNA levels of COX-2, iNOS, MMP-13, and ADAMTS-4 compared to the siLRP4 group, while the mRNA levels of Col-2 and Aggrecan increased (**Figure 8E**). Finally, we treated transfected HNPCs with different concentrations of digoxin for 24 hours and measured the mRNA levels of Col-2 and Aggrecan. As depicted in **Figure 8F**, after LRP4 knockdown, the stimulatory effect of digoxin on the upregulation of Col-2 and Aggrecan mRNA expression was significantly inhibited, but this effect was reversed after restoring LRP4.

## 4 Discussion

IVDD is a significant contributor to back pain and disability worldwide (2, 31). It involves an imbalance between anabolic and catabolic processes (32). Progressive degradation of the ECM ensues in the course of IVDD, accompanied by an augmentation of cellular apoptosis and a concomitant exacerbation of inflammatory responses (32, 33). This multifaceted pathological process underscores the necessity of comprehensive therapeutic strategies that address these pivotal aspects of IVDD (33).

Pharmacological approaches for IVDD have been extensively investigated, but the development of effective therapeutics remains challenging (7, 34) chiefly due to the complexity of the mechanisms involved in IVDD. Moreover, most current pharmacological treatments for disc degeneration mainly focus on managing symptoms and reducing inflammation (35, 36). Consequently, the discovery of drugs that can modify the disease course is of paramount importance. Our study sheds light on the potential of digoxin in addressing this unmet need.

The pathophysiology of IVDD is significantly influenced by the TNF-α and NF-κB signaling pathways (36, 37). TNF-α, a potent proinflammatory cytokine, is responsible for the initiation and progression of disc degeneration by stimulating the NF-κB pathway, which promotes inflammation, ECM degradation, and apoptosis. TNF-α upregulates key mediators of inflammation and catabolic activities, such as COX-2, iNOS, and IL-1β (8, 13). The results of our study indicate that digoxin may have anti-inflammatory properties, as evidenced by its ability to inhibit the upregulation of COX-2 and iNOS induced by TNF-α, as well as its ability to reduce the expression of IL-1β in the intervertebral disc. These findings suggest that digoxin has the potential to mitigate the inflammatory response associated with IVDD.

Aggrecan and Col-2 are crucial components of the ECM in the intervertebral disc, responsible for maintaining hydration, compressive resistance, and structural integrity (38, 39). Dysregulation of Aggrecan and Col-2 contributes to the breakdown of the disc (40). The loss of these constituents is a major feature of IVDD. The degradation of Col-2 and Aggrecan is attributed to the upregulation of MMPs, specifically MMP-13, and A Disintegrin and Metalloproteinase with Thrombospondin Motifs (ADAMTS), specifically ADAMTS-4/5. Our findings revealed that digoxin treatment inhibited the upregulation of MMP-13 and ADAMTS4/5 induced by TNF-α, leading to restoration of the expression of Col-2 and Aggrecan, indicating its potential role in inhibiting ECM degradation in disc degeneration. Besides matrix degradation metalloproteinase, it is reported digoxin alone exerts strong anabolic promotive effects in the cartilage repair process. Given this importance, the present study found digoxin has a dual effect on IVDD by inhibiting catabolic destruction and promoting anabolism.

Apoptosis, or programmed cell death, is a prominent feature of disc degeneration. Increased apoptotic activity in NP cells contributes to the loss of cellularity and ECM components in the disc (41). When TNF-α is present in excessive amounts, as is often the case in pathological conditions like disc degeneration, it can lead to an increase in Bax and a decrease in Bcl-2, tipping the balance toward apoptosis. Caspase-3 operates as a pivotal executioner in the apoptosis pathway, becoming activated in the aftermath of apoptotic initiation (13). Our findings revealed that digoxin treatment downregulated TNF-α-induced apoptosis in NP cells, as evidenced by the reduced expression of the pro-apoptotic protein Bax and decreased cleaved caspase-3 levels. This suggests a potential role of digoxin in inhibiting apoptosis and preserving the viability of NP cells in disc degeneration.

Throughout the course of IVDD, TNF-α plays a crucial role in regulating the inflammatory response and ECM metabolism by activating the NF-κB pathway. As a transcription factor, NF-κB modulates the expression of a wide range of genes associated with inflammation, such as COX-2, iNOS, and MMPs. Upon TNF-α stimulation, NF-κB is triggered and migrates into the cell nucleus, where it associates with specific DNA sequences, consequently promoting the synthesis and release of inflammatory mediators, provoking ECM degradation, and ultimately accelerating IVDD. Our investigation indicates that digoxin diminishes the phosphorylation of p65 protein post TNF-α treatment and significantly impedes the nuclear translocation of p65, highlighting the suppression of TNF-α induced activation of the NF-κB pathway by digoxin.

The ERK1/2 and AKT signaling pathways play crucial roles in regulating ECM synthesis and anabolism in NP cells (42). Activation of these pathways promotes the expression of anabolic factors, including Aggrecan and Col-2, and enhances ECM deposition. Interestingly, our study demonstrated that digoxin treatment activated the ERK1/2 and AKT pathways, leading to increased mRNA expression of Aggrecan and Col-2, suggesting its potential role in promoting ECM anabolism and counteracting the catabolic processes in disc degeneration. Furthermore, the ERK1/2 and AKT signaling pathways can modulate apoptosis in NPCs through diverse mechanisms. Digoxin, through its ability to activate the ERK1/2 and AKT pathways, potentially counteracts cellular apoptosis. Nevertheless, additional experiments are necessary to explore the reciprocal relationship between digoxin’s demonstrated anti-apoptotic capabilities and these two pathways.

LRP4 acts as a transmembrane protein implicated in myriad cellular processes, demonstrated to engage in the Agrin-mediated homeostasis of chondrocytes. Antecedent reports have depicted LRP4 as a therapeutic target for digoxin in osteoarthritis treatment (22), while our research reveals a correlation between its dysregulation and IVDD. As the severity of IVDD intensifies, a parallel decline in the expression of LRP4 in the NP tissues is observed. When LRP4 is knockdown, the capacity of digoxin to counteract the inflammation and ECM degradation mediated by the TNF-α/NF-κB pathway weakens, and its ability to stimulate ECM anabolism via the ERK1/2 and AKT pathways is obstructed. However, these effects were reversed after restoring LRP4 expression, suggesting that LRP4 could potentially serve as a latent target for digoxin in protecting against IVDD.

**Figure 9:**
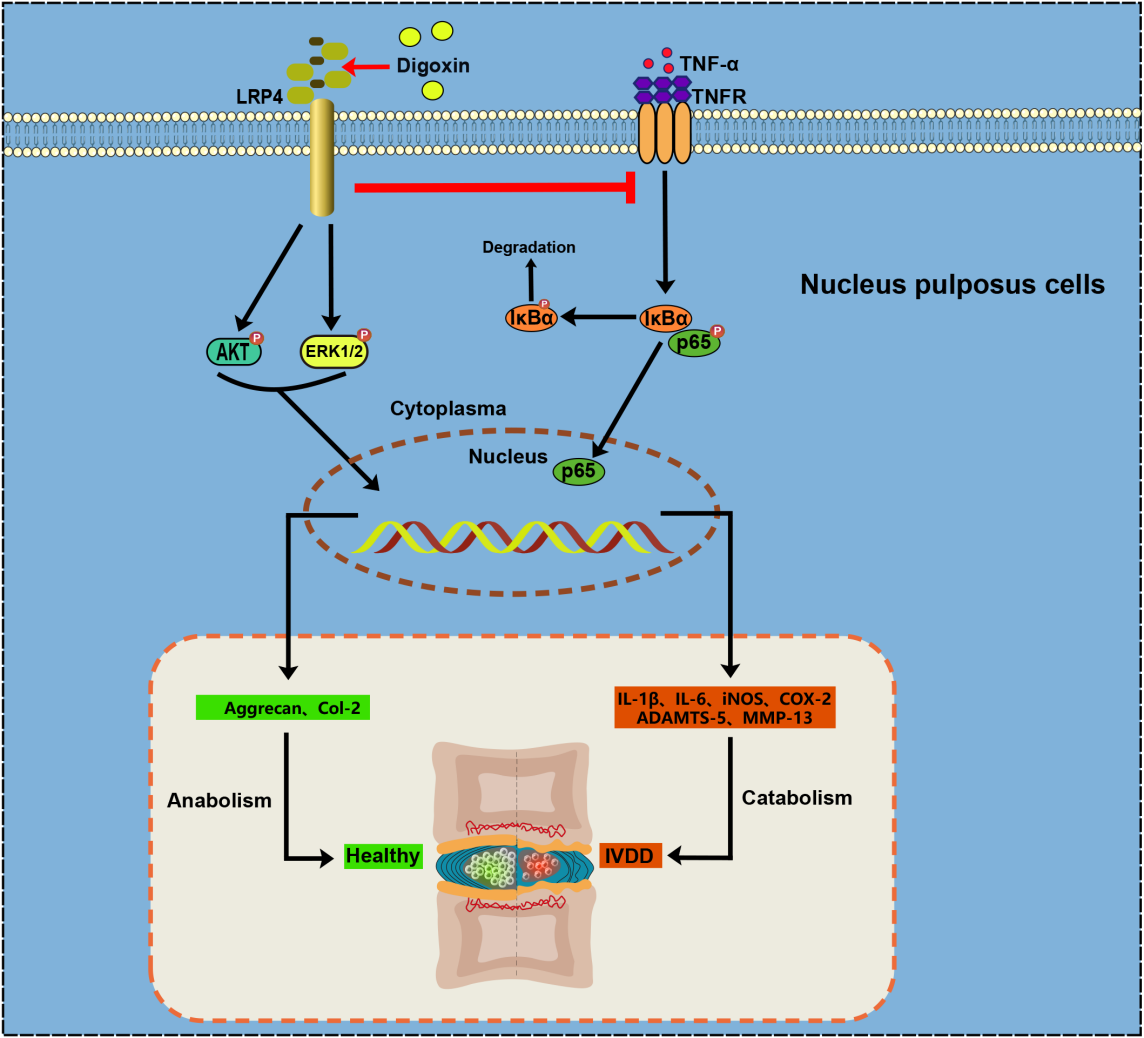
Proposal for Digoxin in intervertebral disc degeneration.

In conclusion, our findings underscore the potential of digoxin as a multifaceted therapeutic agent for IVDD (**Figure 9**). It appears capable of modulating inflammation, promoting ECM anabolism, and decreasing apoptosis. The suggested involvement of LRP4 in these processes provides a new direction for further research into the molecular mechanisms underlying IVDD and its treatment​.

## Data availability statement

The original contributions presented in the study are included in the article/**Supplementary Material**. Further inquiries can be directed to the corresponding authors.

## Ethics statement

The studies involving humans were approved by Medical Ethics Committee at Qilu Hospital of Shandong University. The studies were conducted in accordance with the local legislation and institutional requirements. The participants provided their written informed consent to participate in this study. The animal study was approved by Animal Experimentation Center of Shandong University. The study was conducted in accordance with the local legislation and institutional requirements.

## Author contributions

QM: Performed the experiments, analyzed data, and draft manuscript. KL: draft manuscript, established animal models. ZL: Analyzed and interpreted the data. JL: Maintained the mice and primary cells. ZT and SQ: collected tissues and achieved MRI scans. JW and LC: Conceived and designed the experiments, draft the manuscript. We confirm that the order of authors listed in the manuscript has been approved by all of us. We further confirm that all authors have read and approved the final version of the manuscript, have agreed to its submission for publication, and accept accountability for all aspects of the work in ensuring that questions related to the accuracy or integrity of any part of the work are appropriately investigated and resolved.

## Supplementary material

Supplementary Figure 1
**(A)** Human nucleus pulposus cells were subjected to treatment with or without 0.1 µM Istaroxime hydrochloride in the presence of 50 nM Digoxin for a duration of 24 hours, following which, the mRNA levels related to COX-2, iNOS, MMP-13, ADAMTS-4, Aggrecan, and Col-2 were evaluated using Real-time PCR. **(B)** Western blot and Real-time PCR analyses were employed to investigate the expression levels of LRP4 in human nucleus pulposus cells of varying Pfirrmann grades (n=6). **(C)** Following euthanasia, rat nucleus pulposus cells were isolated for ex vivo cultivation in the control group, needle surgery group, and the group receiving 50 nM Digoxin treatment post-needle surgery. Subsequently, Western blot analyses were conducted to ascertain the changes in LRP4 expression within rat nucleus pulposus cells (n=6). The statistical analysis employed ordinary one-way ANOVA to assess the differences between groups, followed by Tukey’s multiple comparisons test as a *post hoc* analysis. The values shown above represent the mean ± SEM **p < 0.01, *** P ≤ 0.001, **** P ≤ 0.0001 versus the comparison group. ns, no significance.
